# Isolation and molecular characterization of newly emerging avian reovirus variants and novel strains in Pennsylvania, USA, 2011–2014

**DOI:** 10.1038/srep14727

**Published:** 2015-10-15

**Authors:** Huaguang Lu, Yi Tang, Patricia A. Dunn, Eva A. Wallner-Pendleton, Lin Lin, Eric A. Knoll

**Affiliations:** 1 Animal Diagnostic Laboratory, Department of Veterinary and Biomedical Sciences, The Pennsylvania State University, University Park, PA 16802.

## Abstract

Avian reovirus (ARV) infections of broiler and turkey flocks have caused significant clinical disease and economic losses in Pennsylvania (PA) since 2011. Most of the ARV-infected birds suffered from severe arthritis, tenosynovitis, pericarditis and depressed growth or runting-stunting syndrome (RSS). A high morbidity (up to 20% to 40%) was observed in ARV-affected flocks, and the flock mortality was occasionally as high as 10%. ARV infections in turkeys were diagnosed for the first time in PA in 2011. From 2011 to 2014, a total of 301 ARV isolations were made from affected PA poultry. The molecular characterization of the Sigma C gene of 114 field isolates, representing most ARV outbreaks, revealed that only 21.93% of the 114 sequenced ARV isolates were in the same genotyping cluster (cluster 1) as the ARV vaccine strains (S1133, 1733, and 2048), whereas 78.07% of the sequenced isolates were in genotyping clusters 2, 3, 4, 5, and 6 (which were distinct from the vaccine strains) and represented newly emerging ARV variants. In particular, genotyping cluster 6 was a new ARV genotype that was identified for the first time in 10 novel PA ARV variants of field isolates.

Avian reoviruses (ARVs) are widespread in nature and are associated with a wide range of diseases affecting various avian species^1^,^2^, including chickens^3^,^4^, pheasants^5^, turkeys^6^,^7^, ducks^8^,^9^,^10^, geese^11^, pigeons^12^, quails^13^,^14^,^15^, raptors^16^, and psittacine birds^17^. However, most clinical diseases from ARV infections are observed in broiler and broiler breeder chickens^18^. In young broilers, the most common clinical syndromes are tenosynovitis, malabsorption syndrome, runting-stunting syndrome (RSS), enteric disease problems, and immunosuppression^19^,^20^,^21^,^22^,^23^. ARV infections in domestic poultry have several economically significant effects. These include increased mortality, a general lack of performance, diminished weight gain, poor feed conversion, an uneven growth rate, reduced marketability of the affected birds, viral arthritis/tenosynovitis, and secondary infections from other viruses or bacteria^2^,^22^.

ARVs belong to the Orthoreovirus genus in the Reoviridae family^24^,^25^. They contain 10 double-stranded RNA (dsRNA) genome segments, including 3 L (large), 3 M (medium), and 4 S (small) size classes based on the segments’ electrophoretic mobility^26^. Research findings have revealed that the sigma C protein encoded by the S1 genome segment is the cell attachment protein and a major antigenic determinant for ARVs; the S1 genome segment of existing chicken ARV strains is well characterized and well conserved in viruses from chickens^27^,^28^,^29^,^30^. Turkey-origin ARV strains circulating in the Midwest US in recent years are antigenically distinct from chicken-origin ARV strains. The turkey-origin ARV strains are considered a separate virus subtype within the Orthoreovirus genus^31^,^32^,^33^.

Newly emerging ARV infections have occurred in Pennsylvania (PA), USA, since 2011 and have since caused major disease and economic losses in the PA poultry industry. A conservative estimate of the costs of these ARV infections in broiler chickens is $23,000/per affected flock (28,000 birds/flock), and a turkey company estimated $3 million in losses in one year. Vaccination against ARV with conventional vaccines prior to the observed outbreaks had been practiced in layer and broiler breeders. However, these conventional vaccines did not appear to confer any protection against field ARV infections. Until this time, turkey breeder flocks had not been administered ARV vaccines. No ARV vaccinations had been practiced in commercial flocks of layers, broilers or turkeys. Since the detection of variant ARV infections in commercial turkey and broiler flocks, the poultry industry has resorted to the vaccination of breeder flocks with killed autogenous ARV vaccines.

ARV infections in broiler chickens and turkeys have been increasingly diagnosed in PA since 2011 and continue to be observed. Between 2011 and 2014, 301 cases (flocks) were confirmed to be ARV infection by virus isolation in our laboratory. Most of the ARV-positive cases involved sick broilers and turkeys with severe arthritis/tenosynovitis, involving multiple joints and tendons of the legs including stifle, hock, and foot pads, with inflammation extending into the surrounding musculature. A high morbidity (20–40%) and mortality (up to 10%) were often present. This paper describes our diagnostic and research findings in the isolation and molecular characterization of these ARV variants from PA, USA.

## Results

### ARV clinical signs and necropsy lesions

**Figure 1:**
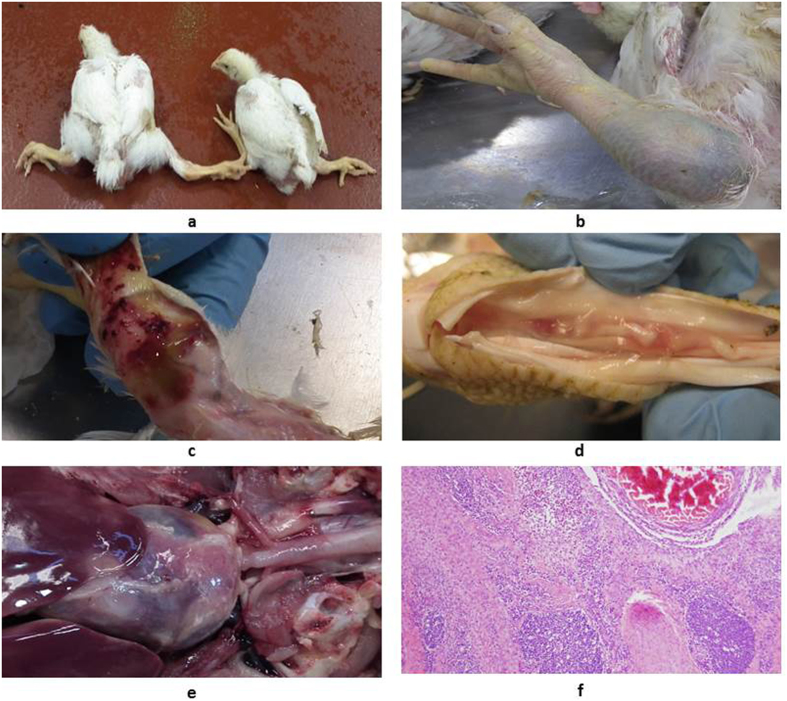
Clinical signs and pathological lesions of avian reovirus infections in broiler chickens. (a) Broiler cases with severe tenosynovitis at 4 weeks of age; (b) Tenosynovitis associated with the entire leg; (c) Swelling, edema, and hemorrhages in the tendons and tendon sheaths; (d) Full-thickness tendon rupture; (e) Lesions of pericarditis; (f) Microscope lesions of chronic lymphocytic plasmacytic tenosynovitis on cross section of tendon, synovium, and associated tissues near the intertarsal joint.

ARV infections have caused significant clinical disease and economic losses in PA poultry since 2011, particularly in broiler chickens and turkeys. ARV infections in turkeys were diagnosed for the first time in PA in June 2011 (Table 1, #54: Reo/PA/Turkey/12883/11). ARV-infected broiler and turkey flocks suffer from severe lameness and splay-leg due to tenosynovitis spanning the femorotibiotarsal and intertarsal joints and plantar metatarsal region and, in some cases, inflammation extending into the surrounding musculature (Fig. 1a,b). Disease onset usually occurred between 2 and 4 weeks of age in broiler flocks and at 10 weeks or greater in turkey flocks. A high morbidity (up to 20% to 40%) of the ARV-infected birds in a flock was commonly observed, and the mortality in the most severe cases was up to 10%. The presence of major gross pathologic lesions included marked swelling, edema, hemorrhages in the tendons and tendon sheaths, and, in more chronic cases, full-thickness tendon rupture with severe hemorrhage (Fig. 1c,d). Pericarditis lesions were also present in some affected birds (Fig. 1e). Microscopically, the predominant inflammatory cell types in the affected tissues were lymphocytes and plasma cells (Fig. 1f).

### ARV isolation and identification

**Figure 2:**
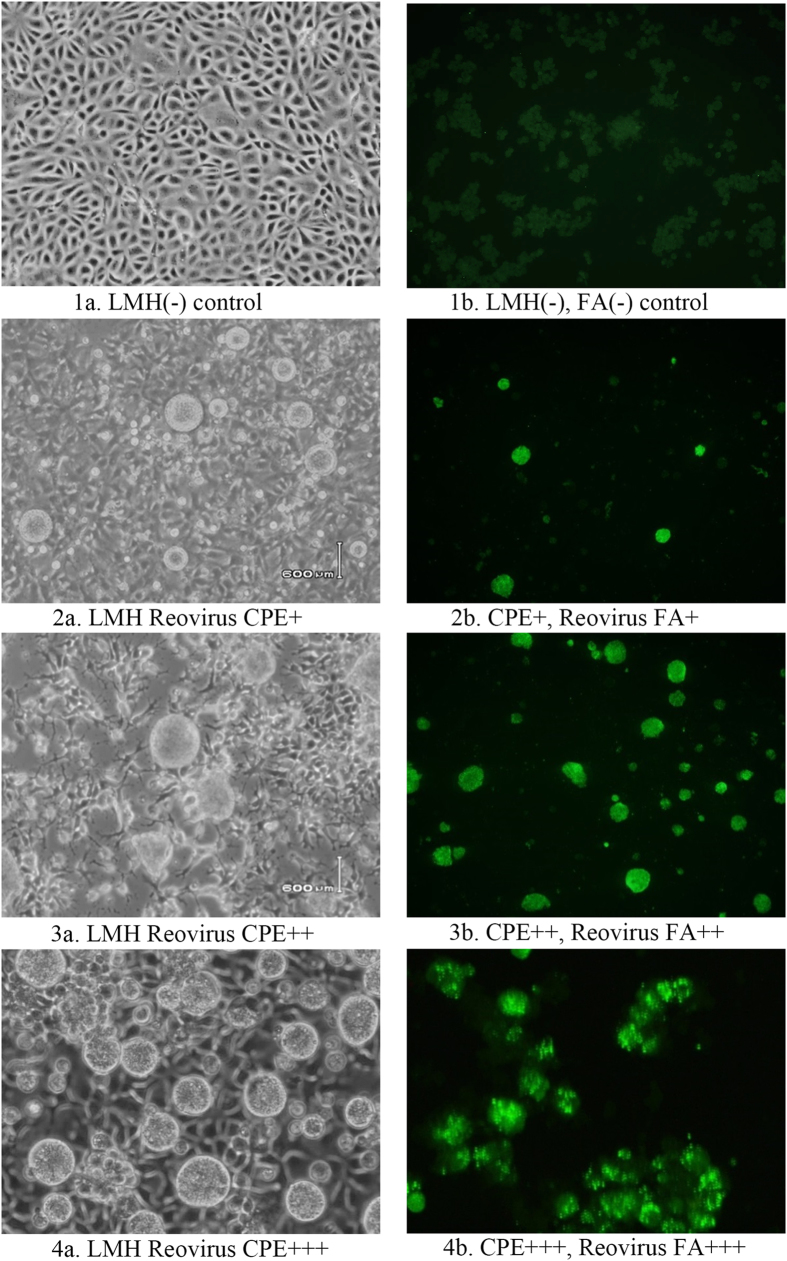
Avian Reovirus (ARV) detection by fluorescent antibody (FA) test on reovirus-infected cytopathic effect (CPE) cells. (1a) Negative control of LMH normal cell cultures; (2a), (3a) and (4a) Giant, or “bloom-like,” CPE cells characteristic to ARV infections in LMH cell cultures; (1b) FA test negative on normal LMH cells; (2b), (3b), and (4b) FA test positives on ARV-infected CPE cells. Note: The FA stained uninfected LMH cell sheet (1b) and ARV-infected cell sheets (2b, 3b, 4b) were harvested from the corresponding LMH cell cultures (1a, 2a, 3a, 4a) in approximately 1 ml of culture media and then prepared on glass slides for the FA test.

In total, 301 ARV field isolates were obtained in our laboratory from 2011 to 2014, mostly from tendons and some from other tissues (hearts, livers, or intestines) of ARV-infected birds. The virus isolations were conducted in LMH (ATCC CRL-2113) monolayer cell cultures (Figs 2, 1a). Of the 301 ARV field isolates, 206 were from broilers, 18 from layers, 63 from turkeys, 7 from chukar partridges, 4 from guinea fowl, 2 from ring-necked pheasants, and 1 from bobwhite quails (Supplement Table 1). Giant, or “bloom-like”, cytopathic effects (CPEs) were characteristic of ARV infections in LMH cell cultures (Figs 2, 1b–d). All of the 301 ARV isolates were confirmed by giant or “bloom-like” CPE-positive cells, which were subsequently stained positive for ARV by the fluorescent antibody (FA) test using a fluorescent ARV antibody (Fig. 2b–d). The incubation periods for CPE varied: the earliest CPE was observed 24 hours post inoculation (pi); the latest was observed, in a few cases, after 4 serial cell passages; and for most ARV-positive cases, CPE was observed 3–5 days pi, within 2–3 serial cell passages.

### RT-PCR and σC gene sequences

In total, 114 ARV field isolates representing broiler, layer, turkey, pheasant, and guinea fowl ARV cases diagnosed between 2011 and 2014 were selected for molecular characterization of the S1 segment of the σC gene (Table 1). Each of the 114 ARV isolates was successfully amplified as a 1088 bp fragment by S1-based RT-PCR using P1/P4 primers^34^. The PCR product was then purified and submitted to the Penn State Genomics Core Facility for S1 gene sequencing.

### Six genotyping clusters of PA ARV field strains

**Figure 3:**
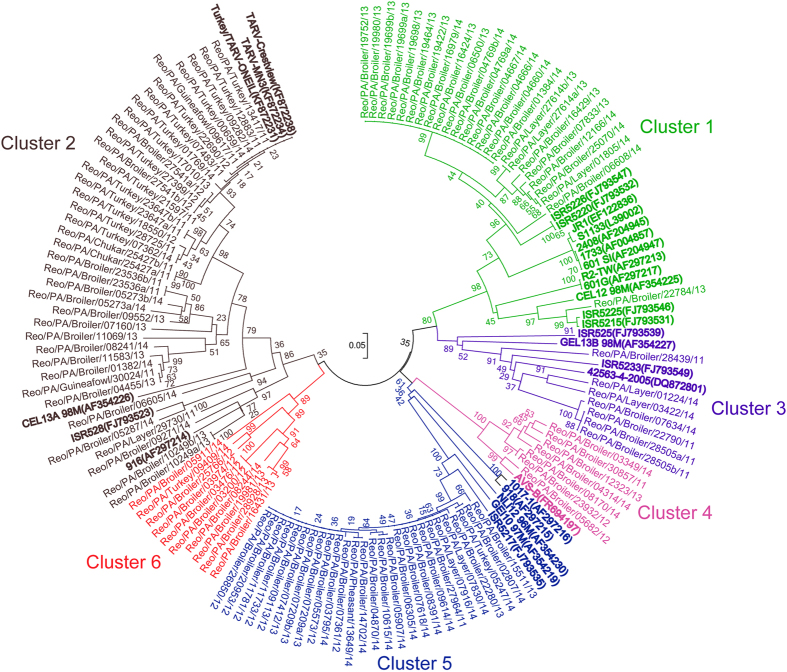
Phylogenetic trees showing 6 genotyping clusters (6 coded colors) of the 114 avian reovirus (ARV) field strains isolated in Pennsylvania of the USA, 2011-2014. The analysis was based on 300 amino acid sequences of σC gene sequences. Branch lengths are proportional to the evolutionary distances between sequences. The scales represent nucleotide substitutions per position. Names of the 28 ARV reference strains retrieved from GenBank are in clusters 1-5 only (bolded to distinguish them from the field strains).

Construction of phylogenetic trees and analysis for conservation of the σC gene S1 segment sequences of the 114 ARV field strains (Table 1) with another 28 reference strain sequences (Supplement Table 2) retrieved from GenBank revealed that the 114 ARV field strains isolated from PA poultry were grouped into 6 genotyping clusters, or genotypes (Fig. 3). Of the 114 field strains in these clusters, 25 (21.93%) were in the same cluster (cluster 1) as the standard ARV vaccine strains (S1133, 1733, 2048); 38 (33.33%) in cluster 2; 7 (6.14%) in cluster 3 and 4; 27 (23.68%) in cluster 5; and 10 (8.77%) in cluster 6 (Table 2; Fig. 3). In particular, genotyping cluster 6, or genotype 6, was identified for the first time in 10 ARV field strains detected in PA poultry, and these strains were novel and distinct from all previously published ARV reference strains.

### Phylogenetic and sequence analysis of σC gene divisions

Pairwise comparison of the prediction of amino acid (aa) sequence (1 to 300) was performed to examine the degree of sequence identity of the homologues of σC genes between the 114 PA ARV field strains and the 28 ARV reference strains retrieved from GenBank (Fig. 3). In general, the aa sequence identities of the σC encoding genes were found to vary dramatically (40% to 100%) among the 114 PA ARV field strains; the aa similarities were less than 60.8% between any 2 of the 6 genotyping clusters; various degrees of differences between the aa similarities within each cluster were observed.

The classification of ARV genotyping clusters and subclusters was based on the bootstrap values (analysis performed with 1000 pseudoreplicates). When the 114 field strains and 28 reference strains were plotted together to build phylogenetic trees (circle tree or linear tree), the circle tree (Fig. 3) was better than the linear tree at clearly illustrating clusters and subclusters.

In genotyping cluster 1 (Fig. 3), the 25 PA ARV field strains shared high sequence identity (71.0–100%), and they were divided into 3 different sub-clusters: sub-cluster 1 was formed by 16 PA broiler strains, sharing 98.4–100% aa identity. Sub-cluster 2 was formed by 5 broiler strains and 3 layer strains, sharing 80.6–97.6% aa identity. All 11 ARV reference strains, including the standard vaccine strains retrieved from GenBank and the remaining 1 PA broiler strain, formed sub-cluster 3. The 24 PA ARV field strains in sub-clusters 1 and 2 shared only 70.6–88.8% aa identities with ARV reference strains and 72.1–75.4% aa identities with the 1 PA ARV broiler strain in sub-cluster 3.

In genotyping cluster 2, the 38 PA ARV field strains shared a wide range (58.8–100%) of aa sequence identity and formed 3 different sub-clusters. Sub-cluster 1 was formed by few chicken-origin strains but included 15 turkey strains, 2 chukar partridge strains, 2 broiler strains, and 1 guinea fowl strain, and they shared 90.8–100% aa identity. Sub-cluster 2 consisted of 11 broiler strains and 1 guinea fowl strain, and they shared 78.6–99.1% aa identity. Sub-cluster 3 included 5 broiler strains, 1 layer strain, and 3 reference strains, and they shared 66.0–100% aa identity.

In genotyping cluster 3, 4 of the 5 broiler strains (except Reo/Broiler/PA/28439/11) and 2 layer strains shared only 77.8–80.5% aa identity with the 4 GenBank reference strains. In cluster 4, all of the 7 PA ARV field strains were broiler-origin, and 6 of the 7 shared high (93.1–99.1%) aa identity; the remaining strain (Reo/PA/Broiler/05682/12) had a high similarity to the AVS-B strain. Cluster 5 consisted of 27 PA ARV field strains, including 23 broiler strains, 2 layer strains, 1 turkey strain, and 1 ring-neck pheasant strain, with high aa sequence identity to each other (85.2–100%), and they were moderately related to the 5 reference strains (59.8–80.8%) in this cluster.

A new genotyping cluster, cluster 6, was identified for the first time in this study. The 10 novel PA ARV field strains, including 9 broiler strains and 1 turkey strain, constructed the new genotyping cluster 6, which was distinct from clusters 1 through 5. The shared aa identity was 71.0–99.9% within cluster 6 but 42.6–60.1% within the other 5 clusters.

### GenBank deposit

The σC gene sequences of 114 ARV field strains characterized by σC genotyping clusters, representing genotypes 1 through 6 detected in PA poultry in the USA, were deposited in GenBank in October (5 of KM #s) of 2014, January (51 of KP #s) and May (58 of KR #s) of 2015 (Table 1).

## Methods

### ARV specimen collection and processing for virus isolation

Tendons and synovial tissues were the preferred specimens for ARV isolation from birds showing clinical signs and lesions consistent with those of ARV infection^1^,^2^,^35^. Other tissues, including heart, liver, and intestine, were also collected in some cases when pericarditis lesions were observed or when clinical signs of poor growth, malabsorption, or maldigestion were present. Necropsy and sample collection were conducted in the necropsy facility at the Animal Diagnostic Laboratory, The Pennsylvania State University, in accordance with guidelines approved by the United States Department of Agriculture (USDA). (http://www.aphis.usda.gov/animal_health/lab_info_services/downloads/NecropsyGuideline.pdf).

Each collected tissue specimen was minced with sterile scissors in a 20 ml sterile plastic container (Cat No. 14310-684, www.vwr.com) and diluted with viral transport medium at a 1:5 (w/v) dilution. The mixture was then placed in a Stomacher bag and homogenized in a Stomacher blender (Model 80, Seward, Ltd., UK) for 2–3 min. Thereafter, the tissue homogenate was transferred to a 15 ml sterile polypropylene conical tube and centrifuged at 1200 rpm for 10 min at 5 °C. Finally, the supernatant was collected and filtered through a 0.45 nm syringe filter to be ready for cell inoculation for ARV isolation.

### Preparation of LMH cell cultures

LMH (ATCC CRL-2113) is a primary hepatocellular carcinoma epithelial cell line developed from the chemical transformation of tumor nodules in the liver of a male leghorn chicken by long-term treatment with diethylnitrosamine^36^. The LMH cell line has an epithelial phenotype and dendritic morphology. LMH cells are highly sensitive to ARV, fowl adenovirus, birnavirus, rotavirus, poxvirus, and other avian viruses tested in our ongoing research studies. LMH cells are cultured routinely in our avian virology lab for the purpose of isolating avian viruses to diagnose infection.

One preparation of LMH cell growth medium consists of 500 ml of DMEM/F-12 50/50 (Dulbecco’s Modified Eagle’s Medium/Ham’s F-12 50/50 Mix, 1X) with L-glutamine and 15 mM HEPES (Corning Cellgro, Ref No. 10-092-CV, USA), 50 ml of fetal bovine serum (FBS), 5 ml of PSA (Pen-Step-Amp) (Cellgro, Ref No. 30-004-CI), and 2.5 ml of gentamicin sulfate (10 mg/ml). The composition of LMH cell maintenance medium is the same as that of the growth medium, except that it contains only 2% (or 10 ml) FBS. The LMH cell culture procedures are, briefly, as follows: (1) A vial of stock LMH cells (1 ml prepared per T-25 cm^2^ flask, at least 1 × 10^6^/viable cells) was taken from a liquid nitrogen tank, placed in a 37 °C water bath for quick thawing, and then centrifuged at 1000 rpm for 10 minutes at 4 °C; the supernatant was discarded. Alternatively, one flask of ongoing LMH cell culture was processed for subculturing at a ratio of 1:4–1:6 per routine cell culture procedure^37^; (2) The cell pellet was resuspended with 1 ml of pre-warmed growth medium and diluted at a ratio of 1:20 (i.e., 1 ml cell suspension, 19 ml growth medium) for the LMH cell subcultures; (3) The cell suspension was dependent on the flasks (e.g., 2.5 ml per T-12.5 cm^2^ flask, 5 ml per T-25 cm^2^ flask, 1.5–2 ml per well on a 6-well cell culture plate, or 1 ml per well on a 12-well plate, which were routinely used for the diagnostic purpose of avian virus isolation in our lab); (4) The LMH cell-seeded flasks were placed in an incubator set at 37 °C with 5% CO_2_. A confluent monolayer was formed within 48–72 hours, depending on the seeding density of the cells. When a monolayer of 75% or greater confluence was formed, the LMH cell flasks were ready to use for specimen inoculation for avian virus isolation. Uninoculated LMH cell flasks served as continuing cell line subcultures for up to 50 or 100 passages. A seed cell flask could be maintained for 1–2 weeks, and the subculture ratio was 1:4–1:8, as for standard cell subculture procedures^37^.

### ARV isolation in LMH cell cultures

T12.5 cm^2^ flasks and 12- or 24-well plates of monolayer LMH cell cultures were mostly used for ARV or other avian virus isolations in our laboratory. LMH growth medium was removed from the cell culture flasks, which were then rinsed with sterile PBS (8.0 g NaCl, 0.2 g KCl, 1.15 g NaH_2_PO_4_, 0.2 g KH_2_PO_4_, 1000 ml d-H_2_O) to remove residual FBS from the cells. The flasks were inoculated with 0.25 ml (for T12.5 flasks) or 0.5 ml (for T25 flasks) of supernatant from each specimen preparation. A negative control cell flask was inoculated with VTM. The inoculated flasks of cells were incubated in a 37 °C incubator for adsorption of the inoculum for 20–30 minutes. LMH maintenance medium (2.5–3.0 ml for a T12.5 flask, 2.0 ml/per well for a 12-well plate, 1 ml/per well for a 24-well plate) was added to the flasks and incubated at 37 °C under 5% CO_2_. The specimen-inoculated monolayers were examined daily for a period of 5–7 days for the development of viral cytopathic effects (CPEs). Two to three serial cell passages were routinely conducted for each specimen to confirm negative results. Positive CPEs by ARV or other common avian enteric virus infections (e.g., adenovirus, rotavirus, herpesvirus, and birnavirus) were generally determined within 1 to 3 cell passages.

### ARV detection by fluorescent antibody (FA) testing

New procedures developed by us, involving FA staining of early CPE cells, were routinely used for early ARV detection in this study. Briefly, these procedures included the following steps: (1) A sample of 1 ml cell culture fluid containing viral CPE cells (without termination of the cell cultures) was taken from a specimen-inoculated cell culture flask when cells were observed to undergo a CPE and be released into the medium from the monolayer; (2) The cell culture fluid sample was centrifuged at 900 rpm to spin down the CPE cells; (3) The medium supernatant was transferred back to the original flask (which continued to be cultured), and the CPE cells were re-suspended in PBS at a ratio of approximately 1:5; (4) The re-suspended CPE cells were placed on a 25 × 75 × 1 mm microscope glass slide (Globe Scientific, Inc., New Jersey, USA), with 0.1–0.2 ml PBS (or 1–2 drops) per sample and a 10–12 mm-diameter round shape for air drying; (5) The slide was fixed with cold (−20 °C) acetone for 10 min, and the sample area was circled with an ink pen or a diamond pencil; (6) The CPE cells were stained with a fluorescently tagged anti-ARV antibody (ID No. 680 VDL 9501, NVSL, Ames, IA, USA), and the slide was placed in a humidity chamber in a 37 °C incubator for 30–40 min in darkness; (7) The fluorescent antibody was removed by gently rinsing from one end of the slide (thereby not dislodging the cells) with PBS, and the slide was then flooded with PBS for 2–3 min for each of 3 washes total; then, the slide was placed side up on paper towel to allow it to air dry (or the slide was placed in a slide holder and placed in a glass slide jar with a stirrer bar on the bottom, the glass jar was filled with PBS until the slides were covered, and the slide jar was placed on a stirrer plate adjusted to a gentle stirring speed for 8–10 min to complete the wash); (8) The slide was mounted with mounting medium (50% PBS buffer, 50% glycerol, pH 8.4) and the stained region was placed under a cover slip for subsequent examination. The slide was kept at room temperature if viewing was to occur within 1 or 2 hours and was otherwise stored in a refrigerator for viewing within 24 hours. CPE cells that were positive for ARV were stained an apple-green color.

Traditional procedures for virus isolation in cell cultures require a significant amount of CPE development ( > 50–70%) and cell culture termination to conduct subsequent virus identification test(s) to confirm a positive isolate. By using our new procedures, particularly for ARV isolation in this project, early virus isolation was achieved for most ARV-positive cases. Because only a small number of early CPE cells (as few as 5–10) were required for ARV-positive confirmation by FA staining, our virus isolation results for ARV diagnosis were made 2–3 days earlier (on average) than the time of developing above 50–70% CPEs for termination of the cell culture plates.

### RNA extraction and RT-PCR

Viral RNA was extracted from the cell culture supernatant using an RNeasy Mini Kit (Cat. No. 74106, QIAGEN, Valencia, CA), following the manufacturer’s instructions. The extracted RNA was used as a template to amplify a 1088 bp fragment from an ARV S1 segment using the published primers P1/P4^34^. The RT-PCR assay was conducted in a 50 μl reaction mixture using a One Step RT-PCR Kit (Cat. No. 210212, QIAGEN, Valencia, CA) containing 10 μl of template RNA, 25 μl of RNase-free water, 10 μl of 5 × Buffer, 2 μL of dNTP mix (10 mM each dNTP), 1 μl of enzyme mix, and 1 μl of each of the two primers. Amplification was performed with the Applied Biosystems 9700 thermal cycler using a reverse transcription step at 50 °C for 30 min. The initial PCR activation step was set at 95 °C for 15 min; then was followed by 94 °C for 30 s, 50 °C for 30 s, and 72 °C for 90 s of each cycle for 38 cycles; and finally was completed with a single cycle of 72 °C for 5 min.

### RT-PCR product purification and sequencing

The ARV S1 segment RT-PCR products were isolated and visualized in an ethidium bromide stained agarose gel. The specific 1088 bp bands were excised and loaded onto the spin columns of a gel extraction kit (Lot No. 04113KE1, Axygen, Tewksbury, MA) using a simple bind/wash/elute procedure. The purified PCR product was measured using a NanoDrop™1000 (Thermo Scientific, Waltham, MA) spectrophotometer and diluted to 40 ng/μl to be used as sequencing templates. All of the samples and P1/P4 primers (1 μM) were submitted to the Penn State Genomics Core Facility for Sanger sequencing using the 3730XL DNA Analyzer (Applied Biosystems, Grand Island, NY).

### Phylogenetic analysis

We used neighbor-joining methods for phylogenetic analysis in this study. The Lasergene 12 Core Suite (DNASTAR, Inc., Madison, WI, USA) was used for Sanger sequencing data assembly, ORF prediction, and nucleotide sequence translation. BLASTN searches were employed to investigate the sequence similarities between the ARV field strains and reference strains in GenBank (http://blast.ncbi.nlm.nih.gov/Blast.cgi). A phylogenetic analysis was performed on the S1 segment (nucleotides 525–1424) of the σC gene (900 bases). The sequence alignments were performed using the ClustalW 1.83 program (http://align.genome.jp). Neighbor-joining and maximum-likelihood (ML) trees were generated, and tree topologies were validated by bootstrap analysis as implemented in the MEGA program (Version 5.0) with absolute distances following 1000 bootstrap replicates^38^.

The methods of clinical and necropsy diagnosis were carried out in accordance with guidelines approved by the United States Department of Agriculture (USDA). (http://www.aphis.usda.gov/animal_health/lab_info_services/downloads/NecropsyGuideline.pdf). All experimental protocols wereapproved by The Pennsylvania State University, The Office for Research Protections.

## Discussion

Although virus isolation is time consuming, it is always preferred in diagnostic virology and is critically important in discovering new viruses or newly emerging field strains or variants. In this study, our newly modified procedures for the early detection of virus in cell cultures were effectively used for the early diagnosis of ARV cases. As soon as a small number of CPE cells were observed in specimen-inoculated cell cultures, a sample of the CPE cells was collected for ARV FA staining to confirm the virus isolation results without terminating the cell cultures. This allowed an earlier diagnosis than traditional virus isolation procedures, which require waiting until 50–70% CPE development. On average, the virus was isolated 2–3 days earlier for most ARV cases by using the new procedure. Furthermore, the new procedure provides clear results because of the use of concentrated CPE cells and allows the simultaneous detection of additional suspected viruses by preparing duplicate slides.

σC is the most variable protein in ARV^39^. It mediates virus attachment to target cells, and antibodies specific for σC neutralize ARV infections^40^,^41^. In this study, our research findings from the phylogenetic analysis of σC gene sequences revealed that the 114 ARV field strains were genetically different and grouped into 6 genotyping clusters, or genotypes (Fig. 3); 90 of the 114 isolates, in clusters 2–6, were field variants and distinct from the standard ARV vaccine strains (S1133, 1733, 2408), which are grouped in cluster 1. More importantly, a novel genotyping cluster (cluster 6) was identified in this study for the first time. The 10 novel ARV field strains detected in PA poultry (9 from broiler chickens, 1 from turkeys) formed the novel ARV genotyping cluster 6, and the strains exhibited high genetic diversity (up to 30% difference from each other).

Within genotyping cluster 1, 24 of the 25 PA ARV field strains formed separate sub-clusters showing differences from the ARV vaccine strain sub-cluster, and the low aa identity (70.6–88.8%) between these sub-clusters indicate that the 24 PA ARV field strains are not identical to the vaccine strains or are possibly vaccine-related field variants. Similarly, aa identity variations between sub-clusters were also observed in genotyping clusters 2, 3, 4, and 5, in which the majority of the PA ARV field strains formed their own sub-clusters, distinguishing them from the ARV reference strains detected elsewhere (the Netherlands, Germany, the USA, and Taiwan) (Fig. 3; S Table 2). Nonetheless, the novel genotyping cluster 6 and the newly emerging field strains or variants in clusters 1 through 5 indicate that ARV revolutionary mutations or re-combinations have occurred or are continuously occurring, which may continue to yield additional ARV field variants or novel strains.

In genotyping cluster 2, the subcluster 1 consisted of 15 turkey strains, 2 chukar strains, 2 broiler strains, and 1 guinea fowl strain detected in PA. These PA ARV field strains had nucleotide homology ranging from 90.8% to 100% with each other and from 92.3% to 99.8% with the 3 MN turkey ARV strains that occurred in 2011^33^, which suggested that these PA ARV strains were likely transmitted from the Midwest turkey-origin strains.

Because each ARV strain contains 10 genome segments of 3 L (large), 3 M (medium), and 4 S (small) size classes^26^, full genome sequencing characterizations can provide more detailed genome information for ARV field strains of interest. By using traditional genome sequencing procedures^42^, we conducted a complete genomic characterization of the PA broiler ARV field strain (Reo/PA/Broiler/05682/12)^43^. Our genome sequencing findings of this broiler ARV revealed that the greatest sequence similarity was observed with the classic AVS-B strain in the S1 segment of the σC gene. The broiler ARV field strain was only moderately similar to the M2 and M3 segments of the AVS-B strain, and the lowest sequence similarity appeared in the most 5’ sequence of the M2 genome segment.

We are currently conducting full-genome sequencing characterization studies on the newly emerging ARV variants and novel strains by using the Next Generation Sequencing (NGS) Illumina MiSeq system^44^, which allows us to determine the locations of mutated genes in the complete sequences of all 10 genome segments. Recent scientific discoveries that resulted from the application of NGS technologies highlight the striking value of using massively parallel platforms for genetic analyses^45^,^46^,^47^,^48^,^49^. These new methods have expanded previously focused readouts from a variety of DNA preparation protocols to the genome-wide scale and have fine-tuned the resolution of these readouts to single base precision. The sequencing of RNA also has advanced and now includes full-length cDNA analyses, serial analysis of gene expression-based methods, and noncoding RNA discovery. Therefore, the application of NGS methodologies to continue this ARV research will yield full genomic sequence information for the newly emerging ARV field variants and novel strains and will enable us to better understand how these novel strains have achieved revolutionary genomic changes.

The most critical control approach for limiting the clinical disease associated with ARV infections is vaccinating breeders appropriately with efficacious vaccines, thereby reducing the potential for vertical transmission and providing progeny with specific maternal antibodies that protect against the current field strains. In addition to the σC gene S1 segment sequencing characterization reported in this study, full genome characterization of the newly emerging ARV field strains will provide more detailed scientific data, allowing us to better understand ARV mutations, re-combinations, and related molecular epidemiology features. These studies will assist in developing effective autogenous killed-virus and live-virus vaccines and other protection strategies.

## Additional Information

**How to cite this article**: Lu, H. *et al*. Isolation and molecular characterization of newly emerging avian reovirus variants and novel strains in Pennsylvania, USA, 2011–2014. *Sci. Rep*. **5**, 14727; doi: 10.1038/srep14727 (2015).

## Supplementary Material

Supplementary Information

## Figures and Tables

**Table 1:** A list of 114 Pennsylvania (PA) avian reovirus (ARV) field strains deposited in GenBank in October (5 of KM #s) of 2014, January (51 of KP #s) and May (58 of KR #s) of 2015, sorted in σC genotyping clusters 1–6.

Serial No.	ARV Isolate Field Strain ID	Sigma-CGenotypingCluster	GenBankAccession No.	SerialNo.	ARV Isolate Field Strain ID	Sigma-CGenotypingCluster	GenBankAccession No.
1	Reo/PA/Broiler/01384/14	1	KR856956	58	Reo/PA/Turkey/21597/11	2	KR856979
2	Reo/PA/Broiler/04660/14	1	KR856959	59	Reo/PA/Turkey/22690/12	2	KP727801
3	Reo/PA/Broiler/04666/14	1	KP727768	60	Reo/PA/Turkey/23647a/11	2	KP727774
4	Reo/PA/Broiler/04667/14	1	KP727767	61	Reo/PA/Turkey/23647b/11	2	KR856967
5	Reo/PA/Broiler/04769a/14	1	KP727766	62	Reo/PA/Turkey/27399/12	2	KR856975
6	Reo/PA/Broiler/04769b/14	1	KR856957	63	Reo/PA/Turkey/28725/11	2	KP727771
7	Reo/PA/Broiler/06500/13	1	KR856953	64	Reo/PA/Broiler/07634/14	3	KR856992
8	Reo/PA/Broiler/06608/14	1	KP727770	65	Reo/PA/Broiler/22790/11	3	KP727787
9	Reo/PA/Broiler/07833/13	1	KR856952	66	Reo/PA/Broiler/28439/11	3	KR856989
10	Reo/PA/Broiler/12166/14	1	KR856961	67	Reo/PA/Broiler/28505a/11	3	KP727786
11	Reo/PA/Broiler/16424/13	1	KP727764	68	Reo/PA/Broiler/28505b/11	3	KR856990
12	Reo/PA/Broiler/16429/13	1	KR856954	69	Reo/PA/Layer/01224/14	3	KP727789
13	Reo/PA/Broiler/16979/14	1	KR856962	70	Reo/PA/Layer/03422/14	3	KP727788
14	Reo/PA/Broiler/19422/13	1	KP727760	71	Reo/PA/Broiler/03349/14	4	KR856994
15	Reo/PA/Broiler/19464/13	1	KP727759	72	Reo/PA/Broiler/04314/14	4	KR856995
16	Reo/PA/Broiler/19698/13	1	KR856955	73	Reo/PA/Broiler/05682/12	4	KP727791
17	Reo/PA/Broiler/19699a/13	1	KP727762	74	Reo/PA/Broiler/08170/14	4	KP727796
18	Reo/PA/Broiler/19699b/13	1	KR856960	75	Reo/PA/Broiler/12323/13	4	KP727793
19	Reo/PA/Broiler/19752/13	1	KP727761	76	Reo/PA/Broiler/23932/12	4	KP727792
20	Reo/PA/Broiler/19980/13	1	KP727763	77	Reo/PA/Broiler/30857/11	4	KP727790
21	Reo/PA/Broiler/22784/13	1	KP727765	78	Reo/PA/Broiler/02807/14	5	KP727807
22	Reo/PA/Broiler/25070/14	1	KR856963	79	Reo/PA/Broiler/03795/14	5	KP727805
23	Reo/PA/Layer/01805/14	1	KR856964	80	Reo/PA/Broiler/04870/14	5	KP727806
24	Reo/PA/Layer/27614/13	1	KP727769	81	Reo/PA/Broiler/05573/12	5	KP727800
25	Reo/PA/Layer/27614b/13	1	KR856958	82	Reo/PA/Broiler/05907/14	5	KR857002
26	Reo/PA/Broiler/01382/14	2	KR856980	83	Reo/PA/Broiler/06305/14	5	KP727809
27	Reo/PA/Broiler/04455/13	2	KP727778	84	Reo/PA/Broiler/07209a/13	5	KR856996
28	Reo/PA/Broiler/05273a/14	2	KR856981	85	Reo/PA/Broiler/07209b/13	5	KR856997
29	Reo/PA/Broiler/05273b/14	2	KR856982	86	Reo/PA/Broiler/07361/12	5	KP727797
30	Reo/PA/Broiler/05287/14	2	KR856986	87	Reo/PA/Broiler/07412/13	5	KP727799
31	Reo/PA/Broiler/06605/14	2	KR856987	88	Reo/PA/Broiler/07618/14	5	KP727810
32	Reo/PA/Broiler/07160/13	2	KR856977	89	Reo/PA/Broiler/08391/14	5	KR857007
33	Reo/PA/Broiler/08241/14	2	KP727782	90	Reo/PA/Broiler/09113/12	5	KP727804
34	Reo/PA/Broiler/09271/14	2	KR856984	91	Reo/PA/Broiler/09614/14	5	KR857003
35	Reo/PA/Broiler/09552/13	2	KP727775	92	Reo/PA/Broiler/10615/14	5	KR857004
36	Reo/PA/Broiler/10249a/13	2	KR856973	93	Reo/PA/Broiler/11733/12	5	KP727803
37	Reo/PA/Broiler/10249b/13	2	KR856966	94	Reo/PA/Broiler/11781/12	5	KP727802
38	Reo/PA/Broiler/11069/13	2	KR856974	95	Reo/PA/Broiler/14702/14	5	KR857005
39	Reo/PA/Broiler/11583/13	2	KR856965	96	Reo/PA/Broiler/15511/13	5	KR857000
40	Reo/PA/Broiler/23536a/11	2	KP727773	97	Reo/PA/Broiler/20953/12	5	KR856999
41	Reo/PA/Broiler/23536b/11	2	KR856971	98	Reo/PA/Broiler/22280/13	5	KR857001
42	Reo/PA/Broiler/27541/12	2	KR856976	99	Reo/PA/Broiler/26850/12	5	KR856998
43	Reo/PA/Broiler/27541a/12	2	KP727776	100	Reo/PA/Broiler/27964/11	5	KP727798
44	Reo/PA/Chukar/25427/11	2	KR856970	101	Reo/PA/Layer/07830/14	5	KP727811
45	Reo/PA/Chukar/25427a/11	2	KP727772	102	Reo/PA/Layer/07916/14	5	KP727812
46	Reo/PA/Guineafowl/09617/11	2	KR856978	103	Reo/PA/Pheasant/13649/14	5	KR857006
47	Reo/PA/Guineafowl/30024/11	2	KR856969	104	Reo/PA/Turkey/05247/14	5	KP727808
48	Reo/PA/Layer/29730/11	2	KR856968	105	Reo/PA/Broiler/03200/12	6	KP727785
49	Reo/PA/Turkey/00659/14	2	KM116024	106	Reo/PA/Broiler/03476/12	6	KP727784
50	Reo/PA/Turkey/01769/14	2	KM116025	107	Reo/PA/Broiler/03974/12	6	KP727783
51	Reo/PA/Turkey/07362/14	2	KR856983	108	Reo/PA/Broiler/05911/14	6	KR857009
52	Reo/PA/Turkey/07483/11	2	KR856972	109	Reo/PA/Broiler/08244/14	6	KR857008
53	Reo/PA/Turkey/09282/14	2	KR856985	110	Reo/PA/Broiler/16431/13	6	KP727794
54	Reo/PA/Turkey/12883/11	2	KM116023	111	Reo/PA/Broiler/19981/13	6	KR856993
55	Reo/PA/Turkey/13417/11	2	KM116022	112	Reo/PA/Broiler/25766/12	6	KR856991
56	Reo/PA/Turkey/17010/13	2	KM116021	113	Reo/PA/Broiler/28928/13	6	KP727795
57	Reo/PA/Turkey/18550/12	2	KP727777	114	Reo/PA/Turkey/09409/14	6	KR856988

**Table 2:** Summary of 114 Pennsylvania (PA) avian reovirus (ARV) field strains, grouped into 6 genotyping clusters based on S1 segment σC gene sequencing characterizations and a total of 301 ARV field isolates obtained from broilers, layers, turkeys, and other avian species in Pennsylvania of the USA, 2011–2014.

Avian	ARV genotyping clusters based on S1 segment σC gene sequences							Sequencing	Total ARV
Species	1	2	3	4	5	6	Total	Not Done	Isolates
Broiler	22	18	5	7	23	9	84	122	206
Layer	3	1	2	0	2	0	8	10	18
Turkey	0	15	0	0	1	1	17	46	63
Chukar	0	2	0	0	0	0	2	5	7
Guinea fowl	0	2	0	0	0	0	2	2	4
Pheasant	0	0	0	0	1	0	1	1	2
Quail	0	0	0	0	0	0	0	1	1
Total	25	38	7	7	27	10	114	187	301
Genotype %	21.93%	33.33%	6.14%	6.14%	23.68%	8.77%	100%		
