# Supplementary material for: Isolation and molecular characterization of newly emerging avian reovirus variants and novel strains in Pennsylvania, USA, 2011–2014
Source: Sci Rep. 2015 Oct 15;5:14727. doi: 10.1038/srep14727 (PMC4606735; doi:10.1038/srep14727)
Supplement: Supplementary Information [file srep14727-s1.pdf]

Isolation and molecular characterization of newly emerging avian reovirus variants and novel strains in Pennsylvania, USA, 2011-2014

Authors: Huaguang Lu*, Yi Tang, Patricia A. Dunn, Eva A. Wallner-Pendleton, Lin Lin, and Eric A. Knoll

*Corresponding author's telephone: 814-863-4369; fax: 814-865-4717; e-mail: hxl15@psu.edu

Supplement Table 1. A list of 301 avian reovirus (ARV) field strains isolated from broilers, layers, turkeys, and other avian species in Pennsylvania, 2011-2014, 114 of them with genotyping clusters based on the S1 segment σC gene sequencing characterizations

Serial No.	ARV Isolate Field Strain ID	Sigma-C Genotyping Cluster	Serial No.	ARV Isolate Field Strain ID	Sigma-C Genotyping Cluster
	**Broilers**				
1	Reo/PA/Broiler/01384/14	1	104	Reo/PA/Broiler/07361/12	ND
2	Reo/PA/Broiler/04660/14	1	105	Reo/PA/Broiler/07361/12	ND
3	Reo/PA/Broiler/04666/14	1	106	Reo/PA/Broiler/07412/13	ND
4	Reo/PA/Broiler/04667/14	1	107	Reo/PA/Broiler/07486/14	ND
5	Reo/PA/Broiler/04769a/14	1	108	Reo/PA/Broiler/08173/14	ND
6	Reo/PA/Broiler/04769b/14	1	109	Reo/PA/Broiler/08197/12	ND
7	Reo/PA/Broiler/06500/13	1	110	Reo/PA/Broiler/08645/12	ND
8	Reo/PA/Broiler/06608/14	1	111	Reo/PA/Broiler/09451/12	ND
9	Reo/PA/Broiler/07833/13	1	112	Reo/PA/Broiler/09460/12	ND
10	Reo/PA/Broiler/12166/14	1	113	Reo/PA/Broiler/09600/12	ND
11	Reo/PA/Broiler/16424/13	1	114	Reo/PA/Broiler/10023/14	ND
12	Reo/PA/Broiler/16429/13	1	115	Reo/PA/Broiler/10076/14	ND
13	Reo/PA/Broiler/16979/14	1	116	Reo/PA/Broiler/10180/12	ND
14	Reo/PA/Broiler/19422/13	1	117	Reo/PA/Broiler/10391/12	ND
15	Reo/PA/Broiler/19464/13	1	118	Reo/PA/Broiler/10441/12	ND

16	Reo/PA/Broiler/19698/13	1	119	Reo/PA/Broiler/10581/12	ND
17	Reo/PA/Broiler/19699a/13	1	120	Reo/PA/Broiler/10646/14	ND
18	Reo/PA/Broiler/19699b/13	1	121	Reo/PA/Broiler/11582/13	ND
19	Reo/PA/Broiler/19752/13	1	122	Reo/PA/Broiler/11732/12	ND
20	Reo/PA/Broiler/19980/13	1	123	Reo/PA/Broiler/11733/12	ND
21	Reo/PA/Broiler/22784/13	1	124	Reo/PA/Broiler/11780/12	ND
22	Reo/PA/Broiler/25070/14	1	125	Reo/PA/Broiler/11781/12	ND
23	Reo/PA/Broiler/01382/14	2	126	Reo/PA/Broiler/11892/12	ND
24	Reo/PA/Broiler/04455/13	2	127	Reo/PA/Broiler/12590/12	ND
25	Reo/PA/Broiler/05273a/14	2	128	Reo/PA/Broiler/12591/12	ND
26	Reo/PA/Broiler/05273b/14	2	129	Reo/PA/Broiler/12602/12	ND
27	Reo/PA/Broiler/05287/14	2	130	Reo/PA/Broiler/14015/12	ND
28	Reo/PA/Broiler/06605/14	2	131	Reo/PA/Broiler/15173/14	ND
29	Reo/PA/Broiler/07160/13	2	132	Reo/PA/Broiler/15573/12	ND
30	Reo/PA/Broiler/08241/14	2	133	Reo/PA/Broiler/16893/14	ND
31	Reo/PA/Broiler/09271/14	2	134	Reo/PA/Broiler/17178/14	ND
32	Reo/PA/Broiler/09552/13	2	135	Reo/PA/Broiler/17418a/13	ND
33	Reo/PA/Broiler/10249a/13	2	136	Reo/PA/Broiler/17614/13	ND
34	Reo/PA/Broiler/10249b/13	2	137	Reo/PA/Broiler/17766/13	ND
35	Reo/PA/Broiler/11069/13	2	138	Reo/PA/Broiler/17818/13	ND
36	Reo/PA/Broiler/11583/13	2	139	Reo/PA/Broiler/18607/14	ND
37	Reo/PA/Broiler/23536a/11	2	140	Reo/PA/Broiler/19134/14	ND
38	Reo/PA/Broiler/23536b/11	2	141	Reo/PA/Broiler/19138/14	ND
39	Reo/PA/Broiler/27541a/12	2	142	Reo/PA/Broiler/19422/13	ND
40	Reo/PA/Broiler/27541b/12	2	143	Reo/PA/Broiler/19821/14	ND
41	Reo/PA/Broiler/07634/14	3	144	Reo/PA/Broiler/19840/13	ND
42	Reo/PA/Broiler/22790/11	3	145	Reo/PA/Broiler/20240/13	ND
43	Reo/PA/Broiler/28439/11	3	146	Reo/PA/Broiler/20294/13	ND
44	Reo/PA/Broiler/28505a/11	3	147	Reo/PA/Broiler/20585/13	ND
45	Reo/PA/Broiler/28505b/11	3	148	Reo/PA/Broiler/20904/12	ND
46	Reo/PA/Broiler/03349/14	4	149	Reo/PA/Broiler/20921/14	ND

47	Reo/PA/Broiler/04314/14	4	150	Reo/PA/Broiler/20953/12	ND
48	Reo/PA/Broiler/05682/12	4	151	Reo/PA/Broiler/21073/14	ND
49	Reo/PA/Broiler/08170/14	4	152	Reo/PA/Broiler/21331/14	ND
50	Reo/PA/Broiler/12323/13	4	153	Reo/PA/Broiler/21784/13	ND
51	Reo/PA/Broiler/23932/12	4	154	Reo/PA/Broiler/22279/13	ND
52	Reo/PA/Broiler/30857/11	4	155	Reo/PA/Broiler/22280a/13	ND
53	Reo/PA/Broiler/02807/14	5	156	Reo/PA/Broiler/22280b/13	ND
54	Reo/PA/Broiler/03795/14	5	157	Reo/PA/Broiler/22790/11	ND
55	Reo/PA/Broiler/04870/14	5	158	Reo/PA/Broiler/22884/14	ND
56	Reo/PA/Broiler/05573/12	5	159	Reo/PA/Broiler/22990/14	ND
57	Reo/PA/Broiler/05907/14	5	160	Reo/PA/Broiler/22999/14	ND
58	Reo/PA/Broiler/06305/14	5	161	Reo/PA/Broiler/23370/14	ND
59	Reo/PA/Broiler/07209a/13	5	162	Reo/PA/Broiler/23370/14	ND
60	Reo/PA/Broiler/07209b/13	5	163	Reo/PA/Broiler/23454a/14	ND
61	Reo/PA/Broiler/07361/12	5	164	Reo/PA/Broiler/23454b/14	ND
62	Reo/PA/Broiler/07412/13	5	165	Reo/PA/Broiler/23536a/11	ND
63	Reo/PA/Broiler/07618/14	5	166	Reo/PA/Broiler/23536b/11	ND
64	Reo/PA/Broiler/08391/14	5	167	Reo/PA/Broiler/23536c/11	ND
65	Reo/PA/Broiler/09113/12	5	168	Reo/PA/Broiler/23932/12	ND
66	Reo/PA/Broiler/09614/14	5	169	Reo/PA/Broiler/23962a/14	ND
67	Reo/PA/Broiler/10615/14	5	170	Reo/PA/Broiler/23962b/14	ND
68	Reo/PA/Broiler/11733/12	5	171	Reo/PA/Broiler/24139/13	ND
69	Reo/PA/Broiler/11781/12	5	172	Reo/PA/Broiler/24272/12	ND
70	Reo/PA/Broiler/14702/14	5	173	Reo/PA/Broiler/24486/13	ND
71	Reo/PA/Broiler/15511/13	5	174	Reo/PA/Broiler/24598/14	ND
72	Reo/PA/Broiler/20953/12	5	175	Reo/PA/Broiler/24598/14	ND
73	Reo/PA/Broiler/22280/13	5	176	Reo/PA/Broiler/24677/12	ND
74	Reo/PA/Broiler/26850/12	5	177	Reo/PA/Broiler/24948/14	ND
75	Reo/PA/Broiler/27964/11	5	178	Reo/PA/Broiler/25070/14	ND
76	Reo/PA/Broiler/03200a/12	6	179	Reo/PA/Broiler/25193/14	ND
77	Reo/PA/Broiler/03200b/12	6	180	Reo/PA/Broiler/25197/14	ND

78	Reo/PA/Broiler/03476/12	6	181	Reo/PA/Broiler/25210/14	ND
79	Reo/PA/Broiler/03974/12	6	182	Reo/PA/Broiler/25345/13	ND
80	Reo/PA/Broiler/05911/14	6	183	Reo/PA/Broiler/25353/13	ND
81	Reo/PA/Broiler/08244/14	6	184	Reo/PA/Broiler/25889/14	ND
82	Reo/PA/Broiler/16431/13	6	185	Reo/PA/Broiler/25983/14	ND
83	Reo/PA/Broiler/19981/13	6	186	Reo/PA/Broiler/25983/14	ND
84	Reo/PA/Broiler/25766/12	6	187	Reo/PA/Broiler/26273/14	ND
85	Reo/PA/Broiler/28928/13	6	188	Reo/PA/Broiler/26427/13	ND
86	Reo/PA/Broiler/01384/14	ND	189	Reo/PA/Broiler/26595/12	ND
87	Reo/PA/Broiler/03200a/12	ND	190	Reo/PA/Broiler/27052/14	ND
88	Reo/PA/Broiler/03260/14	ND	191	Reo/PA/Broiler/27052/14	ND
89	Reo/PA/Broiler/03262/14	ND	192	Reo/PA/Broiler/27333/14	ND
90	Reo/PA/Broiler/03476/12	ND	193	Reo/PA/Broiler/27333/14	ND
91	Reo/PA/Broiler/03476/12	ND	194	Reo/PA/Broiler/27541a/12	ND
92	Reo/PA/Broiler/03476/12	ND	195	Reo/PA/Broiler/27541b/12	ND
93	Reo/PA/Broiler/03974/12	ND	196	Reo/PA/Broiler/27541c/12	ND
94	Reo/PA/Broiler/04030/14	ND	197	Reo/PA/Broiler/27964/11	ND
95	Reo/PA/Broiler/04315/14	ND	198	Reo/PA/Broiler/28028/14	ND
96	Reo/PA/Broiler/05260/14	ND	199	Reo/PA/Broiler/28114/14	ND
97	Reo/PA/Broiler/05573/12	ND	200	Reo/PA/Broiler/28437/11	ND
98	Reo/PA/Broiler/05686/12	ND	201	Reo/PA/Broiler/28439/11	ND
99	Reo/PA/Broiler/05864a/13	ND	202	Reo/PA/Broiler/28544/14	ND
100	Reo/PA/Broiler/05864b/13	ND	203	Reo/PA/Broiler/28549/14	ND
101	Reo/PA/Broiler/05982/12	ND	204	Reo/PA/Broiler/28638/13	ND
102	Reo/PA/Broiler/06504/13	ND	205	Reo/PA/Broiler/28861/12	ND
103	Reo/PA/Broiler/07160/13	ND	206	Reo/PA/Broiler/29028/12	ND
	**Layers**				
1	Reo/PA/Layer/01805/14	1	10	Reo/PA/Layer/06387/14	ND
2	Reo/PA/Layer/27614/13	1	11	Reo/PA/Layer/08549/12	ND
3	Reo/PA/Layer/27614b/13	1	12	Reo/PA/Layer/11848/13	ND

4	Reo/PA/Layer/29730/11	2	13	Reo/PA/Layer/16979a/14	ND
5	Reo/PA/Layer/01224/14	3	14	Reo/PA/Layer/16979b/14	ND
6	Reo/PA/Layer/03422/14	3	15	Reo/PA/Layer/24533/13	ND
7	Reo/PA/Layer/07830/14	5	16	Reo/PA/Layer/24947a/14	ND
8	Reo/PA/Layer/07916/14	5	17	Reo/PA/Layer/24947b/14	ND
9	Reo/PA/Layer/03120/14	ND	18	Reo/PA/Layer/06453/13	ND
	**Turkeys**				
1	Reo/PA/Turkey/00659/14	2	33	Reo/PA/Turkey/16922/14	
2	Reo/PA/Turkey/01769/14	2	34	Reo/PA/Turkey/18131a/14	ND
3	Reo/PA/Turkey/07362/14	2	35	Reo/PA/Turkey/18131b/14	ND
4	Reo/PA/Turkey/07483/11	2	36	Reo/PA/Turkey/18378/14	ND
5	Reo/PA/Turkey/09282/14	2	37	Reo/PA/Turkey/18421a/14	ND
6	Reo/PA/Turkey/12883/11	2	38	Reo/PA/Turkey/18421b/14	ND
7	Reo/PA/Turkey/13417/11	2	39	Reo/PA/Turkey/18648/14	ND
8	Reo/PA/Turkey/17010/13	2	40	Reo/PA/Turkey/19707/13	ND
9	Reo/PA/Turkey/18550/12	2	41	Reo/PA/Turkey/19766/14	ND
10	Reo/PA/Turkey/21597/11	2	42	Reo/PA/Turkey/20711/13	ND
11	Reo/PA/Turkey/23647a/11	2	43	Reo/PA/Turkey/20925/14	ND
12	Reo/PA/Turkey/23647b/11	2	44	Reo/PA/Turkey/21006/14	ND
13	Reo/PA/Turkey/27399/12	2	45	Reo/PA/Turkey/21455a/13	ND
14	Reo/PA/Turkey/28725/11	2	46	Reo/PA/Turkey/21455b/13	ND
15	Reo/PA/Turkey/22690/12	2	47	Reo/PA/Turkey/21602a/14	ND
16	Reo/PA/Turkey/05247/14	5	48	Reo/PA/Turkey/21602b/14	ND
17	Reo/PA/Turkey/09409/14	6	49	Reo/PA/Turkey/21629/13	ND
18	Reo/PA/Turkey/03063a/12	ND	50	Reo/PA/Turkey/21743/14	ND
19	Reo/PA/Turkey/03063b/12	ND	51	Reo/PA/Turkey/21810/14	ND
20	Reo/PA/Turkey/03063/12	ND	52	Reo/PA/Turkey/22342a/13	ND
21	Reo/PA/Turkey/03302/14	ND	53	Reo/PA/Turkey/22342b/13	ND
22	Reo/PA/Turkey/07431/14	ND	54	Reo/PA/Turkey/22428a/14	ND
23	Reo/PA/Turkey/09071/12	ND	55	Reo/PA/Turkey/22428b/14	ND

24	Reo/PA/Turkey/11487/12	ND	56	Reo/PA/Turkey/22463/14	ND
25	Reo/PA/Turkey/12242/12	ND	57	Reo/PA/Turkey/22690a/12	ND
26	Reo/PA/Turkey/12378/13	ND	58	Reo/PA/Turkey/22690b/12	ND
27	Reo/PA/Turkey/12571/12	ND	59	Reo/PA/Turkey/22800/14	ND
28	Reo/PA/Turkey/13518a/12	ND	60	Reo/PA/Turkey/25564/13	ND
29	Reo/PA/Turkey/14894/12	ND	61	Reo/PA/Turkey/26347/11	ND
30	Reo/PA/Turkey/16090a/11	ND	62	Reo/PA/Turkey/31215/11	ND
31	Reo/PA/Turkey/16090b/11	ND	63	Reo/PA/Turkey/31223/11	ND
32	Reo/PA/Turkey/16690/13	ND			
	**Other Avian Species**				
1	Reo/PA/Chukar/25427a/11	2	8	Reo/PA/GuineaFwl/09617/11	2
2	Reo/PA/Chukar/25427b/11	2	9	Reo/PA/GuineaFwl/30024/11	2
3	Reo/PA/Chukar/13811/12	ND	10	Reo/PA/GuineaFwl/14614/12	ND
4	Reo/PA/Chukar/22243/13	ND	11	Reo/PA/GuineaFwl/30024/11	ND
5	Reo/PA/Chukar/22244/13	ND	12	Reo/PA/Pheasant/13649a/14	5
6	Reo/PA/Chukar/30179a/11	ND	13	Reo/PA/Pheasant/13649b/14	ND
7	Reo/PA/Chukar/30179b/11	ND	14	Reo/PA/Quail/21105/12	ND

Supplement Table 2. A list of 28 avian reovirus (ARV) reference strains retrieved from GenBank

Serial No.	Name of ARV reference Strains	Avian Species	Country or Region Origin	Year	GenBank Accession Number	S1 segment σC Genotyping Cluster
1	ISR5225	Chicken	Israel	2006	FJ793546	1
2	ISR5215	Chicken	Israel	2007	FJ793531	1
3	ISR5226	Chicken	Israel	2007	FJ793547	1
4	ISR5220	Chicken	Israel	2007	FJ793532	1
5	ISR528	Chicken	Israel	2005	FJ793523	2
6	ISR5217	Chicken	Israel	2007	FJ793535	5

7	ISR5223	Chicken	Israel	2007	FJ793549	3
8	ISR525	Chicken	Israel	2005	FJ793539	3
9	GEL12 98M	Chicken	Germany	1998	AF354225	1
10	GEI10 97M	Chicken	Germany	1997	AF354219	5
11	GEL13A 98M	Chicken	Germany	1998	AF354226	2
12	GEL13B 98M	Chicken	Germany	1998	AF354227	3
13	NLI12 96M	Chicken	Netherland	1996	AF354230	5
14	601G	Chicken	Taiwan	1992	AF297217	1
15	R2-TW	Chicken	Taiwan	1992	AF297213	1
16	601SI	Chicken	Taiwan	1992	AF204947	1
17	916	Chicken	Taiwan	1992	AF297214	2
18	918	Chicken	Taiwan	1992	AF297215	4
19	1017-1	Chicken	Taiwan	1992	AF297216	5
20	JR1	Chicken	USA	2006	EF122836	1
21	2048	Chicken	USA	1983	AF204945	1
22	1733	Chicken	USA	1983	AF330703	1
23	S1133	Chicken	USA	1973	AF330703	1
24	AVS-B	Chicken	USA	2005	FR694197	4
25	42563-4/2005	Chicken	USA	2005	DQ872801	3
26	TARV-MN3	Turkey	MN, USA	2011	KF872234	2
27	TARV-O'NEIL	Turkey	MN, USA	2011	KF872231	2
28	TARV-Crestview	Turkey	MN, USA	2011	KF872238	2
